# Spontaneous Resolution of Recurrent Pleural Effusion in Atraumatic Splenic Rupture

**DOI:** 10.7759/cureus.40232

**Published:** 2023-06-10

**Authors:** Sushan Gupta, Danish Thameem

**Affiliations:** 1 Internal Medicine, Carle Foundation Hospital, Champaign, USA; 2 Pulmonary and Critical Care Medicine, Carle Foundation Hospital, Champaign, USA

**Keywords:** infectious mononucleosis syndrome, exudative pleural effusion, spontaneous splenic rupture, splenic rupture, recurrent pleural effusion

## Abstract

Spontaneous splenic rupture is an uncommon cause of acute-onset left-sided pleural effusion. It is often immediate with a high preponderance for recurrence, sometimes even requiring splenectomy. We report a case of spontaneous resolution of recurrent pleural effusion presenting a month after the initial atraumatic splenic rupture. Our patient was a 25-year-old male without significant medical history who was taking Emtricitabine/Tenofovir for pre-exposure prophylaxis. He presented to the pulmonology clinic for left-sided pleural effusion, diagnosed in the emergency department a day prior. He had a history of spontaneous grade III splenic injury one month before, where he was diagnosed with cytomegalovirus (CMV) and Epstein-Barr virus (EBV) co-infection on polymerase chain reaction (PCR) testing and was managed conservatively. The patient underwent thoracentesis in the clinic, which showed exudative lymphocyte predominant pleural effusion and no malignant cells. The remainder of the infective workup was negative. He was readmitted two days later with worsening chest pain, and imaging revealed re-accumulation of pleural fluid. The patient declined thoracentesis, and a chest X-ray was repeated a week later, showing worsening pleural effusion. The patient insisted on continuing conservative management, and he was seen a week later with a repeat chest X-ray that showed near resolution of pleural effusion. Splenomegaly and splenic rupture can lead to pleural effusion due to posterior lymphatic obstruction, which can be recurrent. There are no current guidelines on management, and treatment options include watchful monitoring, splenectomy, or partial splenic embolization.

## Introduction

Unilateral pleural effusion is common in clinical practice with a broad differential diagnosis. A low oncotic pressure, increased pulmonary capillary pressure or permeability, and lymphatic obstruction can all lead to pathological fluid accumulation in the pleural cavity [1]. Identification of the underlying etiology is vital for therapeutic and prognostic purposes. The most common causes of unilateral left-sided pleural effusion include infection, malignancy, hepatic hydrothorax, or portal hypertension, especially in the context of cirrhosis and splenomegaly [1]. Recurrent unilateral pleural effusion due to splenic rupture is rare and has only been described to present simultaneously [2-4]. Moreover, guidelines on managing recurrent pleural effusion post-splenic rupture are lacking, and the treatment mainly depends on patient and physician preference. We report a case of spontaneous resolution of persistent pleural effusion presenting after almost a month of atraumatic splenic rupture.

## Case presentation

**Figure 1:**
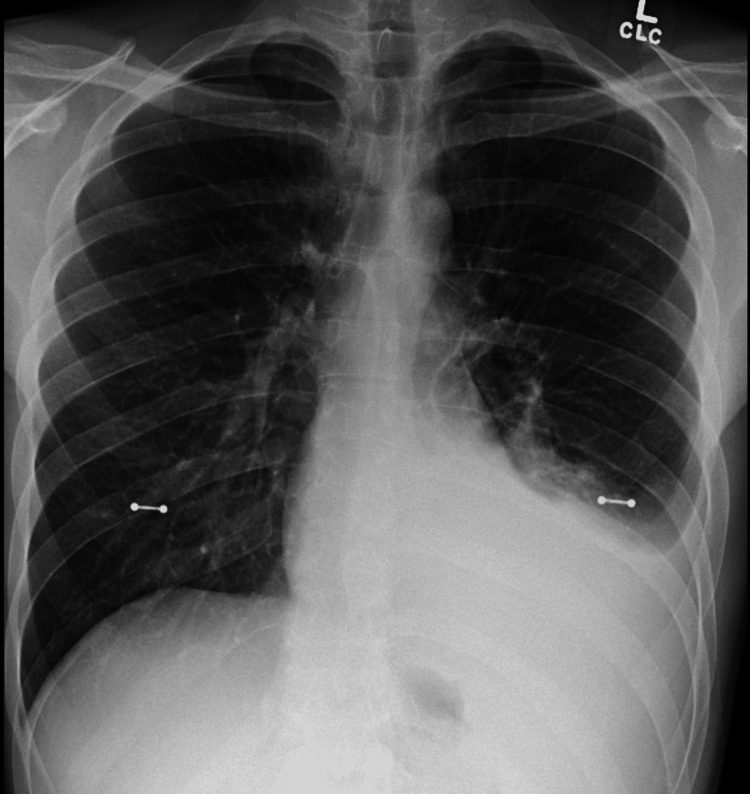
Chest X-ray showing left-sided pleural effusion.

Our patient is a 25-year-old male with no significant medical history who presented to the pulmonology clinic after being admitted to the emergency department a day earlier for left-sided chest pain and was diagnosed with left-sided moderate pleural effusion (Figure 1).

A review of symptoms was negative for fever, cold, cough, orthopnea, shortness of breath, and weight gain. He did endorse taking Emtricitabine/Tenofovir for pre-exposure prophylaxis. He was otherwise a healthy, active PhD student with occasional alcohol use history limited to one to two beers a week. He has had regular testing for sexually transmitted diseases, including HIV, syphilis, hepatitis B and C, and gonorrhea, which have been negative. He was a lifetime nonsmoker and denied recreational drug use. Travel and family history was noncontributory.

Our patient was recently admitted to the hospital a month ago with an episode of localized left upper quadrant pain radiating to the shoulder, with nausea and vomiting. He denied trauma or participation in contact sports at that time. Complete blood count on admission showed a drop in hemoglobin from 11.3 mg/dL two days back to 7.6 mg/dL on admission, with a normal total and differential white blood cell count (Table 1).

**Table 1:** Complete blood count study three days before admission and upon admission. WBC, white blood cell

Laboratory test	Result value (three days before admission)	Result value (on admission)	Reference range
Hemoglobin (g/dL)	11.3	7.6	12.0-18.0
Hematocrit (%)	34.4	24.1	37.0-51.0
Platelet (10^3^/microL)	210	152	140-400
WBC (10^3^/microL)	10.13	5.27	4.00-11.00
Absolute neutrophil count (10^3^/microL)	6.08	1.83	1.60-7.70

**Figure 2:**
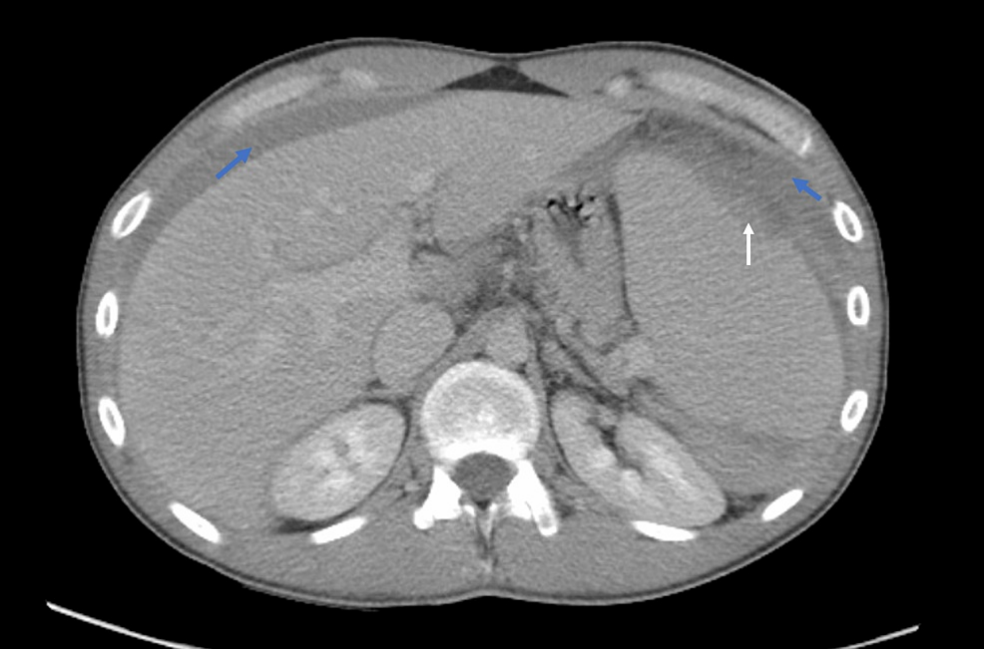
CT scan of the abdomen showing the area of splenic laceration (white arrow) and hemoperitoneum around the spleen and the liver (blue arrow). CT, computed tomography

The complete metabolic panel, including liver and renal function tests, was normal. Infective workup was positive for cytomegalovirus (CMV) and Epstein-Barr virus (EBV). The rest of the workup, including blood cultures and HIV and syphilis testing, was negative. The chest X-ray performed on admission was normal. Initial ultrasound abdomen did not reveal acute pathology. Due to persistent pain, and a drop in hemoglobin, a CT scan of the abdomen and pelvis with contrast was performed that showed the presence of significant splenomegaly, two linear lucencies traversing the superior and superolateral spleen, suspicious for splenic laceration (Grades 3-4 splenic injury) with moderate free fluid in the pelvis compatible with extensive hemoperitoneum (Figure 2).

These findings were confirmed on CT angiography, which did not additionally reveal any active blush or a portal venous thrombus. He received fluid resuscitation and two units of packed red blood cells on admission. General surgery and interventional radiology were consulted, and they recommended conservative treatment with resuscitation and serial hemoglobin monitoring. He remained stable throughout his hospital stay and was discharged three days later with outpatient general surgery follow-up.

A month later, he was readmitted to the emergency department (ED) for left-sided chest pain for two weeks. He was diagnosed with new onset left-sided pleural effusion. He denied any other constitutional symptoms on admission. CT abdomen and pelvis showed persistent stable perisplenic hematoma. Blood work performed in the ED was normal, including stable hemoglobin and WBC counts and normal renal and liver function. The patient insisted on outpatient management of his pleural effusion. As he was hemodynamically stable, maintaining saturation above 94% on room air, he was discharged and visited us in the clinic the next day.

During the current visit, the patient was comfortable and hemodynamically stable. The physical examination was consistent with decreased air entry in the left lung base. The rest of the physical examination was unremarkable. He underwent thoracentesis, and 950 mL of serosanguinous pleural fluid was removed. Pleural fluid labs showed a pH of 7.5, protein 5.1 mg/dL (serum protein 7.2 mg/dL), and lactate dehydrogenase (LDH) of 1,252 mg/dL. Pleural fluid cell differential was lymphocyte dominant (41%). Cytology showed macrophages and reactive mesothelial cells and was negative for malignancy. Infective workup was negative, including pleural fluid aerobic anaerobic, fungal, and mycobacterial cultures. A repeat chest X-ray post-thoracentesis in the clinic showed improvement in the size of pleural effusion and no pneumothorax. Due to the likely post-viral reactive vs. inflammatory cause of pleural effusion, he was managed conservatively with the plan to repeat imaging in two weeks.

**Figure 3:**
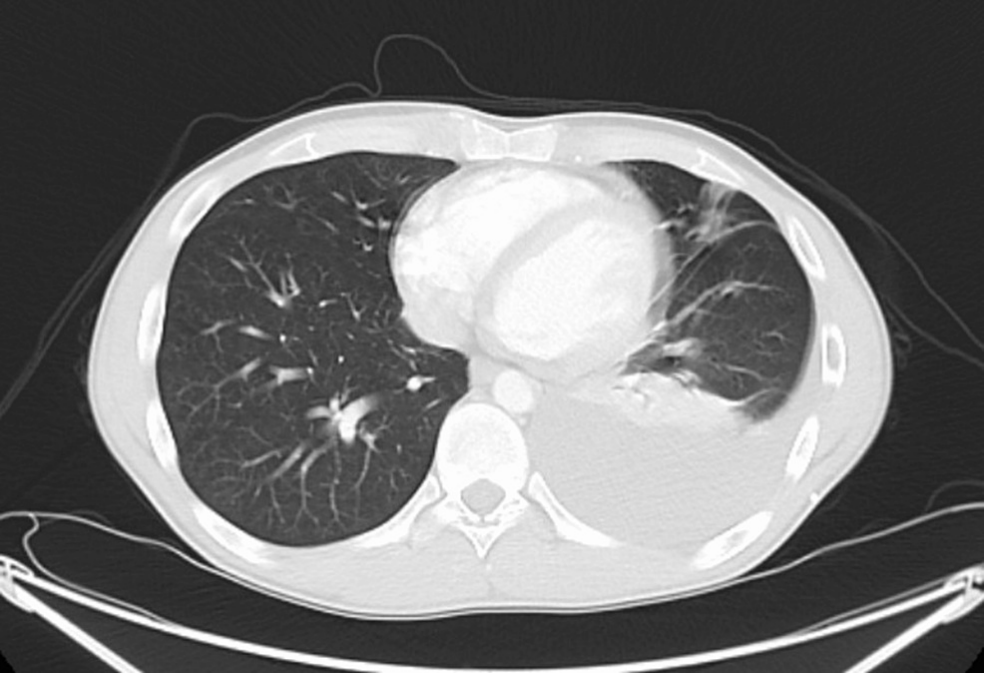
CT scan of the chest showing left-sided pleural effusion. CT, computed tomography

However, the patient got admitted to the ED two days after our visit for persistent left-sided chest pain. He continued to remain hemodynamically stable. A CT pulmonary embolus (CTPE) study in the ED showed a left-sided pleural fluid collection redevelopment with atelectasis in the left lower lobe and no pulmonary embolism (Figure 3).

**Figure 4:**
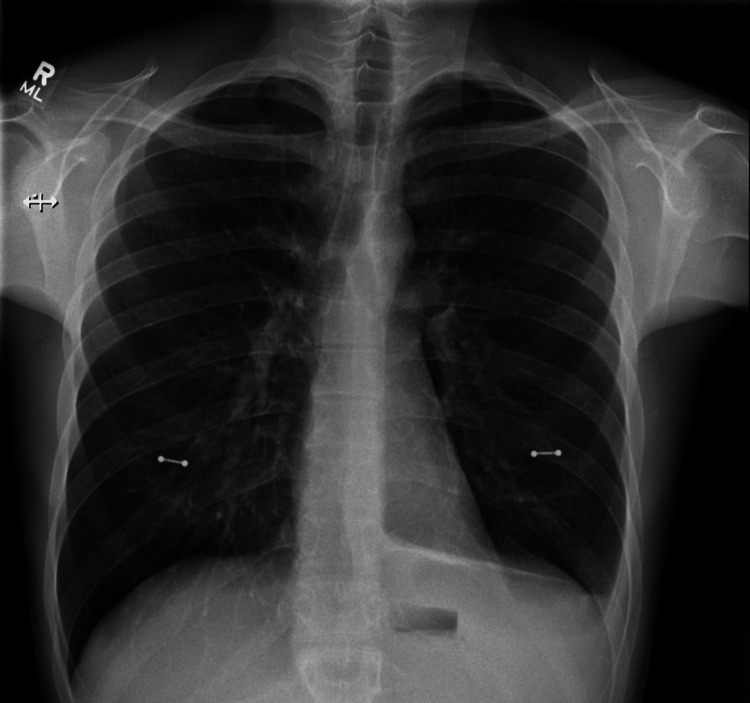
Chest X-ray showing spontaneous near resolution of left-sided pleural effusion.

The patient refused thoracentesis and was discharged after follow-up with a pulmonologist. Repeat chest X-ray during the follow-up visit in one week showed increased left-sided pleural effusion. He again declined thoracentesis and preferred to continue conservative treatment. We repeated another chest X-ray a week later, which showed near resolution of the pleural effusion (Figure 4).

The patient reported improvement in symptoms and preferred further follow-up only if he had any recurrence of symptoms.

## Discussion

Spontaneous splenic rupture with hemorrhage is a rare and life-threatening complication of infectious mononucleosis, seen in 0.1% to 0.5% of cases [5]. Pleural effusion secondary to splenic rupture is rare, especially with no history of splenic trauma. In our review, this is the only case report of spontaneous resolution of persistent pleural effusion a month after the initial presentation of atraumatic splenic rupture.

Neoplastic disorders, especially hematological malignancies, including non-Hodgkin lymphoma, myeloproliferative disorders, and acute myelogenous leukemias, are the most common causes of atraumatic splenic rupture [6]. Viral infections, like infectious mononucleosis, CMV, HIV, and dengue fever, are seen in approximately 15% of cases [6]. In approximately 5% of cases, protozoan infections, predominantly plasmodium species in tropical countries, have been associated with atraumatic splenic rupture [6,7]. *Babesia microti*, a tick-borne parasite, can also present with splenomegaly and spontaneous splenic rupture [8]. It is predominantly seen in the Northeast and upper Midwest (Wisconsin and Minnesota) of the United States and transmitted by infected Ixodes tick bite or rarely by blood transfusion and transplacental spread [9]. Our patient denied travel to endemic areas or places likely to be infested with ticks. Moreover, since he had already tested for infectious mononucleosis, we did not perform further workup for Babesia infection.

Atraumatic splenic rupture constitutes more than 80% of cases with spontaneous splenic rupture seen with infectious mononucleosis [5]. Our patient had a grade III splenic laceration with extensive hemoperitoneum on initial presentation. The management of splenic laceration is based on hemodynamic stability and signs of active bleeding [10]. Clinical examination, especially signs of hypovolemia, is vital in these cases, as it may provide an initial clue to inadvertent active intraperitoneal blood loss. Our patient was hemodynamically stable with additional imaging, including CT angiography, negative for active blush or signs of active bleeding. He was subsequently managed non-operatively with close hemodynamic monitoring and serial hemoglobin checks.

Chest X-ray on imaging at the time of his admission did not reveal any pleural effusion. He was readmitted a month later with exudative moderate-sized pleural effusion. Pneumonia and malignancy often constitute the majority of cases of unilateral exudative pleural effusion. However, the absence of constitutional symptoms, lack of malignant cells on cytology, and a negative infective workup, including normal WBC counts and pleural fluid cultures, likely suggested reactive or inflammatory etiology. Noninfective causes of unilateral pleural effusion have a broad differential, including pulmonary infarction, thromboembolism, collagen vascular diseases, and subphrenic abscess, which are easy to exclude from history, imaging, and lab findings [1].

Splenic injury is a rare differential of left-sided pleural effusion. Koehler and Jones described the first three cases of left-sided pleural effusion in patients with splenic hematoma in the 1980s [2]. In a retrospective study by Kulaylat et al., 4.1% of pediatric splenic trauma patients developed left-sided pleural effusion [3]. The incidence was higher with high-grade splenic injuries. In another case series by Lupien and Sauerbrei involving 16 patients with splenic trauma, left-sided pleural effusion was seen in seven patients [11].

The underlying pathophysiology of left-sided pleural effusion in splenomegaly and splenic trauma is likely multifactorial. Lower left rib fractures and left lung atelectasis in patients with splenic trauma might be significant contributing factors to the development of pleural effusion in these patients [3]. Exudative pleural effusion in atraumatic splenic rupture and splenomegaly could be explained by possible direct compression of posterior lymphatics by an enlarged spleen/hematoma or increased subphrenic permeability due to sympathetic activation associated with splenic trauma leading to accumulation of perisplenic fluid into the pleural cavity [4]. The timing of pleural effusion post-splenic trauma is almost immediate in all case reports [2-4,11]. Our patient presented with pleural effusion a month after spontaneous splenic rupture. Repeat CT abdomen on admission showed stable, persistent splenomegaly and perisplenic hematoma. The reason for delayed presentation is unclear; however, prolonged lymphatic compression and sympathetic activation are likely.

Re-accumulation of pleural fluid has been described in patients with splenic trauma. The initial cases of pleural effusion with splenic hematoma described by Koehler and Jones were also resistant to thoracentesis, eventually needing splenectomy [2]. Recurrent re-accumulation of pleural fluid post-splenic injury was also reported in a study by Warren and Gibbons [12]. In the study by Kulaylat et al., pleural effusion resolved within two weeks of trauma, while the intrasplenic hematoma resolved over months [3]. Our patient refused any invasive management after the initial thoracentesis and opted for conservative management. While no strong evidence exists to guide management in these cases, splenectomy and partial splenic embolization have been described for recurrent pleural effusion seen with splenomegaly of varying etiologies [2,12,13]. The pleural effusion in our patient almost entirely resolved spontaneously after the initial recurrence, suggesting physicians can consider watchful monitoring in these cases after discussing with the patient.

## Conclusions

In conclusion, physicians should remain cognizant of splenic hematoma as a rare differential in patients with isolated left-sided pleural effusion of unclear etiology. The management should be individualized, and options include watchful monitoring, splenectomy, and partial splenic embolization. Our patient was on regular follow-up and showed spontaneous resolution of pleural effusion after the initial recurrence.
